# Thymol-Nanoparticles as Effective Biocides against the Quarantine Pathogen *Xylella fastidiosa*

**DOI:** 10.3390/nano13071285

**Published:** 2023-04-06

**Authors:** Francesca Baldassarre, Daniele Schiavi, Serena Ciarroni, Vincenzo Tagliavento, Angelo De Stradis, Viviana Vergaro, Gian Paolo Suranna, Giorgio Mariano Balestra, Giuseppe Ciccarella

**Affiliations:** 1 Department of Biological and Environmental Sciences, UdR INSTM of Lecce University of Salento, Via Monteroni, 73100 Lecce, Italy; 2 Institute of Nanotechnology, CNR NANOTEC, Consiglio Nazionale Delle Ricerche, Via Monteroni, 73100 Lecce, Italy; 3 Department of Agriculture and Forest Sciences (DAFNE), University of Tuscia, Via S. Camillo de Lellis, Snc, 01100 Viterbo, Italy; 4 Phytoparasites Diagnostics (PhyDia) s.r.l., Via S. Camillo de Lellis, Snc, 01100 Viterbo, Italy; 5 Institute for Sustainable Plant Protection, CNR—IPSP, Consiglio Nazionale delle Ricerche, Via Amendola 165/A, 70126 Bari, Italy; 6 Department of Civil, Environmental, Land, Building Engineering and Chemistry (DICATECh), Politecnico di Bari, Via Orabona 4, 70125 Bari, Italy

**Keywords:** thymol, CaCO_3_ nanocrystals, biocides, plants protection, *Xylella fastidiosa*

## Abstract

Quarantine pathogens require the investigation of new tools for effective plant protection. In particular, research on sustainable agrochemicals is the actual challenge. Plant extracts, essential oils, and gels are natural sources of efficient biocides, such as aromatic secondary metabolites. Thymol is the major phenolic constituent of thyme and oregano essential oils, and it can inhibit many pathogenic microbes. Thymol nanoparticles were obtained through adsorption on CaCO_3_ nanocrystals, exploiting their carrier action. High loading efficiency and capability were reached as verified through UV and TGA measurements. We report the first study of thymol effect on *Xylella fastidiosa,* conducing both fluorometric assay and in vitro inhibition assay. The first test confirmed the great antibacterial effect of this compound. Finally, an in vitro test revealed an interesting synergistic action of thymol and nanocarriers, suggesting the potential application of thymol-nanoparticles as effective biocides to control *Xylella fastidiosa* infection.

## 1. Introduction

Plants are constantly exposed to several pathogenic microorganisms, which affect crops’ productivity and food quality in different agrifood compartments. The current response is the massive agrochemical utilization. The term agrochemicals regard a wide range of compounds, including fungicides, insecticides, herbicides, fertilizers, and plant growth stimulants. These products provide a lot of benefits in crop management, but they could have toxic effects toward not-target living organisms in the soil and in the waters [1,2]. The effects on human health are numerous, from cancer to obesity, as supported by several human epidemiological investigations and experimental animal studies [3,4]. Therefore, the actual challenge in agricultural sciences is the investigation of new strategies to control plant pathogens, reducing the dependency on conventional agrochemicals. In the last years, the exploitation of nanotechnologies led to the development and application of agronanochemicals, which provide controlled and targeted release of active substances, bypassing adverse effects [5,6]. Furthermore, the recent boost comes from natural ingredients, exploiting the ability of plants to produce several metabolites with high antimicrobial effect and intrinsic plant defence mechanisms [7,8]. Aromatic secondary metabolites, such as phenols, phenolic acids, quinones, flavones, flavonoids, tannins, and coumarins, can inhibit both Gram-positive and Gram-negative bacteria and many pathogenic fungi [9]. Plant extracts, essential oils, and gels are natural sources of these efficient biocides, which could provide different applications from agriculture to biomedicine [10]. Thymol (2-isopropyl-5-methyl phenol) is the major phenolic constituent of thyme (*Thymus vulgaris*) and oregano (*Origanum vulgare*) essential oils, and it has been shown great antimicrobial activity. It acts by affecting cells membrane structure and permeability, thus binding bacterial proteins [11,12,13]. Thymol has been extensively investigated to inhibit the proliferation of food fungi, wood decay agents, molds, crop pests, and insects [14,15]. It has been also exploited to control food spoilage and extend shelf life of food products as an alternative to synthetic preserving food products, thanks to its great antioxidant activity [16] However, thymol’s potential in agrifood applications is limited by its poor water solubility, which decreases bioavailability and antimicrobial properties. Its bioactivity is also compromised by susceptibility to heat and volatile character. Furthermore, its antimicrobial activity needs the penetration of microorganism cells by membranes depolarization; this effect takes place thanks to utilization of high concentration [12]. All these drawbacks seriously restrict thymol real investigation and application as a biocide. In recent years, nanoencapsulation of thymol has been investigated to enhance its stability and bioavailability. Exploited materials do not act only as protective shells, but they allow a great interaction with target cells and a gradual release in them [17]. Different systems and materials have been applicated for thymol, including silica colloids, chitosan particles, and cyclodextrins [18,19,20]. Hence, nanogel, nanoparticles, and nanoemulsions were studied for optimizing the usage of thymol as a biocide [19,21,22,23]. This is the first report about the investigation of thymol against the quarantine pathogen *Xylella fastidiosa*. The effective action of several polyphenols is known. However, among these, thymol is not included [24]. *Xylella fastidiosa* is one of the most aggressive and global quarantine pathogens, infecting hundreds of plant species. Its pathogenic effect is due to invasion and obstruction of xylematic vessels, forming biofilm and using many specific sap-feeding vectors for the transmission between plant hosts. Since 2013, it seriously affected olive trees southern Italy, changing the landscape and the regional economy [25]. The linked pathology is known as olive quick decline syndrome (OQDS), and it is caused by the strain “*De Donno*” ST53 of the subspecies *pauca* of this bacterium (XfDD), and there is not a resolutive cure [26]. Chemical nanotechnology can play a decisive role in the fight against this phytopathogen, considering previous results about the nanoformulation of the conventional biocide, fosetyl-Al [27]. Furthermore, calcium carbonate nanocarriers showed an interesting interaction with the integrity of the cell wall of XfDD, perturbing cells vitality with membrane detachment and spheroid particles production [28]. This nanomaterial showed, also, a great affinity to phenols, as demonstrated for caffeic acid and pomegranate peel extract [10,28].

We have investigated thymol in in vitro tests on XfDD, improving its antibacterial activity through the adsorption on calcium carbonate nanocrystals (nanoCaCO_3_). In this report, the adsorption of thymol on nanoCaCO_3_ was studied by tuning initial phenol concentration and quantifying it both through direct and indirect methods. A preliminary fluorescent test on *X. fastidiosa* was performed to evaluate the potential action of this compound on bacteria vitality. In vitro growth assay has shown the bactericidal effect of thymol-nanoparticles, comparing them with free thymol and empty nanoCaCO_3_.

## 2. Materials and Methods

### 2.1. Materials

Calcium chloride dehydrate 99.99% (CaCl_2_·2H_2_O), sodium hydrogen carbonate (NaHCO_3_), 2-Isopropyl-5-methylphenol ≥98.5% (thymol), ethanol 96%, and PYE broth components were purchased from Sigma Aldrich (Milan, Italy). LIVE/DEAD ^®^BacLight™ kit (MolecularProbes) was purchased from Thermo Fisher Scientific Inc. (Waltham, MA, USA), Xpert Fast Probe 2X Mastermix was purchased from GRiSP, Lda. (Porto, Portugal).

### 2.2. Thymol-Nanoparticles Preparation and Characterization

Thymol-nanoparticles (Thy-Np) were produced trough physical adsorption on nanoCaCO_3_ nanocrystals. NanoCaCO_3_ were synthesized, as previous described [29]. Thymol (Thy) adsorption was carried out by adding, drop by drop, an ethanolic solution of phenolic compound to an aqueous solution (pH 7.5) of nanoCaCO_3_ (fixed quantity of 100 mg), reaching the selected final concentrations (2–5–25 mg/mL). The nanoCaCO_3_ suspension was sonicated for 20 min prior loading. The physical adsorption was carried out mixing overnight the suspension at RT, in the dark. The feed concentrations were chosen to overcome thymol solubility in water (0.098 g in 100 g at 25 °C), and a water/alcohol ratio of 4/1 was maintained.

Nanoparticles were collected following centrifugation (6000 rpm for 10 min), and supernatants were stored for subsequent UV-vis analyses. After three washings, Thy-Np was dried in a stove at 50 °C. Washing solutions were stored for subsequent quantification.

Adsorption efficiency (AE) and adsorption capacity (AC) were calculated using the following equations [30,31,32,33]:

(1) $$\%adsorption\;efficiency=\frac{\left[loading\;solution\right]-[supernatant]}{\left[loading\;solution\right]}⨯100$$

(2) $$\%adsorption\;capacity=\frac{{mg}_{adsorbed\;Thy}}{{mg}_{nanoCaCO_{3}}}⨯100$$

where [loading solution] is the initial thymol concentration to which nanocrystals are exposed, [supernatant] is the concentration of free thymol after adsorption experiment, mg_adsorbed Thy_ is the estimated quantity of thymol on nanocrystals surface after washing, and mg_nanoCaCO_3__ is the exposed quantity of nanocrystals.

These data were evaluated both through indirect and direct methods using two different experimental techniques. The first provided the quantification of free thymol in reaction supernatants, and the second provided the destructive analysis of nanoparticles powder. Supernatants quantification was performed by spectrophotometric analysis, recording UV-Vis absorption spectra at 276 nm by a Varian-Cary 500 spectrophotometer. The unknown concentration was obtained referring to a standard curve using thymol solutions at known concentrations in the range of 25–0.15 mg/mL and fitting the line through Origin software (Abs values have been multiplied by dilution factor). Washing solutions were also analysed to quantify thymol molecules, which were weakly adsorbed on nanocrystals. AE was determined by subtraction quantifying, and not adsorbed molecules, into the supernatants. Adsorbed thymol quantity was determined from AE, and the subtraction of thymol mg of washing solutions were calculated with regard to mg_adsorbed Thy_ and AC. The exclusion of weak adsorption allowed us to determinate the exact thymol adsorption capacity on nanocrystals. These data were confirmed by the direct method, exploiting thermogravimetric analysis (TGA) on sample powder (5 mg). TGAs were carried out on a TA Instruments Q600 instrument. A nitrogen flow of 100 mL min^−1^ was set, and a 10 °C/min heating rate was applied, as reported previously [34]. TGA allowed us to quantify adsorbed Thy, as well as recording weight loss % over temperature, observing thermal events of Thy-Np (after washings), Thy and Np (nanoCaCO_3_ alone). Thymol analysis allowed us to determine thermal evaporation of free molecules. Our carriers are inorganic nanomaterials, so it is possible to clearly distinguish thermal events of nanoCaCO_3_ from those of adsorbed organic molecule on Thy-Np.

FT-IR measurements were recorded on a JASCO 4200 spectrophotometer in attenuated total reflectance (ATR) mode.

The morphological analysis of Thy-Np was conducted with scanning electronic microscopy (SEM). A drop of sample was placed on silicon support and dried at room temperature and then was viewed under a SEM MERLIN ZEISS, with a FEG source, at an accelerating voltage of 20 kV, using short exposure time (a few tens of seconds). Instrument software was exploited to determine average diameter of dried Thy-Np. The average diameter (+/− Standard Deviation) has been calculated, analysing five images. These images were taken randomly on representative fields of Thy-Np samples. In total, 100 nanoparticles were analyzed. Excel software was used for average calculations.

Hydrodynamic diameter and polydispersity index measurements were performed through dynamic light scattering analysis (DLS) using the instrument Nano ZS90 (Malvern Instruments, Malvern, UK).

### 2.3. Xylella Fastidiosa Strain

The strain CFBP 8402 of *Xylella fastidiosa* subsp. *pauca* was used in this work. The strain was firstly isolated in symptomatic olive trees in 2014 in the Apulia region (Italy). The bacterium was maintained on BCYE plates at 28 °C and subcultured every 20 days [35].

### 2.4. Fluorescent Assay (Live/Dead Cell Viability Assay)

To evaluate thymol effect on XfDD cells’ vitality, a water suspension of 1 × 10^8^ cells were treated with thymol solutions at different concentrations (0.1–0.25–0.5 mg/mL) for 0–1–2–24 h of incubation time at 38 °C. The treatments were subjected to a differential fluorescent staining by the LIVE/DEAD ^®^BacLight™ (MolecularProbes; Waltham, Massachusetts, USA) viability kit to assess the vitality of bacteria cells. The kit contains two nucleic acid dyes, SYTO 9 and propidium iodide (PI), which allow us to distinguish live cells with intact plasma membranes (green) from dead bacteria with compromised membranes (red). The bacteria–thymol suspensions were incubated at room temperature for 15 min in the dark in a solution of equal volumes of the two stains. Photomicrographs were taken on a Nikon E800 microscope using a fluorescein isothiocyanate (480/30 excitation filter, DM505 dichroic mirror, 535/40 emission filter) and tetramethylrhodamine isocyanate (546/10 excitation filter, DM575 dichroic mirror, 590 emission filter) fluorescence filter sets.

### 2.5. In Vitro Inhibition Assay

To evaluate the antimicrobial properties of the proposed compounds, an in vitro assay was performed on XfDD, following the protocol described by Baldassarre et al. [28]. An amount of 10 μL of a bacterial suspension made from a fresh culture of XfDD, adjusted at OD_600_ = 0.8, was put in sterilized tubes with 1 mL of PYE broth, previously amended with Thy and nanoCaCO_3_ in order to reach the final concentrations of 0.125, 0.25, 0.5, and 1 mg/mL, while Thy-Np was added in order to reach the final concentration of 0.25, 0.5, 1, and 2 mg/mL, since the loaded Thy correspond to 50% in the final Thy-Np formulation. An amount of 1 mg/mL streptomycin sulphate and PYE broth, alone, were used as controls. Tubes were kept under continuous orbital agitation at 28 °C. From each replicate, 100 μL of bacterial suspension were taken after seven and fourteen days post-inoculation (dpi) in order to quantify the presence of XfDD by a real time Taqman PCR [36]. The quantification protocol was performed using a Xpert Fast Probe 2X Mastermix and following the manufacturer’s instructions (GRiSP, Lda. Porto, Portugal). Corresponding cycle threshold (Ct) values of the same thesis were mediated and converted to bacterial concentration (CFU/mL) thanks to the previously obtained calibration curve. For each sample, three technical replicates were made (*n* = 41). The experiment was repeated twice.

#### Statistical Analysis

Collected data from each time point of the inhibition assay were statistical analysed by performing one-way analysis of variance (ANOVA). Assumptions were checked (normal distribution, homogeneity of variances, and homoscedasticity, as well as data independence). Statistical significance of means was studied with Fisher’s LSD post hoc test. *p*-values less than 0.05 were considered significant, while *p*-values of less than 0.01 were considered highly significant. Statistical analyses were performed using XLSTAT 2020.4 (Addinsoft, France).

## 3. Results and Discussion

### 3.1. Thymol Nanoparticles

Calcium carbonate is a very porous material that can entrap different chemicals and, for this feature, it is exploited in several applications, from bioremediation to drug delivery [37]. NanoCaCO_3_ have a great surface/volume ratio, allowing their use as nano-sponges to load high drug concentrations. Hydrophobic molecules have shown a great affinity to nanoCaCO_3_. Our recent results about pomegranate peels extract adsorption demonstrated that, among many compounds, including citrate, sugars and amino acids, polyphenols are those most stably adsorbed on a nanocrystals surface [10,28]. This entrapment efficiency was confirmed for thymol. Adsorption efficiency and capacity are resumed in Table 1 by tuning the initial loading concentration. Adsorption efficiency derived from thymol supernatants quantification. Adsorption capacity considered the quantity of thymol in washing waters, eliminating the non-specific adsorption contribution. The experiments were performed, maintaining fixed nanoCaCO_3_ quantity and choosing feeding concentrations to overcome thymol solubility in water (0.098 g in 100 g at 25 °C). This feature allowed us to promote capillary force of CaCO_3_ pores on dissolved thymol. However, the first experiment, using a final concentration of 2 mg/mL (twice that its solubility), did not allow thymol loading. Thymol loading was found to increase initial concentration to 5 mg/mL, reaching an AE of 22%. This adsorption condition led to a weak entrapment with the complete release of thymol after the two-water washing. Thymol adsorption and retention efficiency were maximized, starting from a concentration of 25 mg/mL that carried out AE and AC of about 90–95%. An increasing trend respect to the initial concentration has been previously found for another phenol, caffeic acid [28]. The high value of AC confirmed the stable physical interaction with nanocrystal surface and the saturation of their pores.

These results are in line with a previous report about thymol loading by hierarchically-structured biogenic silica particles [18]. However, inorganic nanomaterials, including ours, provided better results than polymeric particles, which encapsulated thymol with low capacity in the range of 2.5 to 10%, starting from very concentred solutions [20,31].

Thy-Np sample referred to the best loading condition (loading solution of 25 mg/mL), which was investigated in the following characterizations and antibacterial tests.

**Figure 1:**
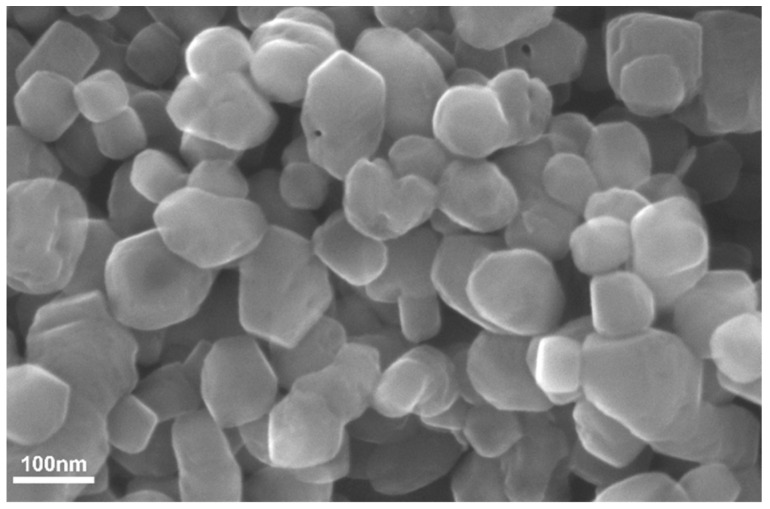
SEM image of Thy-Np.

Thymol adsorption on nanocrystals was also highlighted by SEM analysis. The Thy-Np image in Figure 1 showed the loss of typical cubic shape of nanoCaCO_3_ with amorphous appearance due to relevant organic molecule presence [10,29].

Thy-Np size was determined by direct evaluation on SEM images of dried particles and by DLS analysis, determining the average hydrodynamic diameter and polydispersity index of Np water suspensions. Dimensions data are resumed in the following Table 2.

Our previous works about nanoCaCO_3_ dimensions reported an average diameter of 76.1 ± 0.9 nm (standard deviation) [38]. The Thy-Np measured diameter has a high standard deviation due to the irregular morphology and polydispersity of the sample, which was confirmed by DLS.

**Figure 2:**
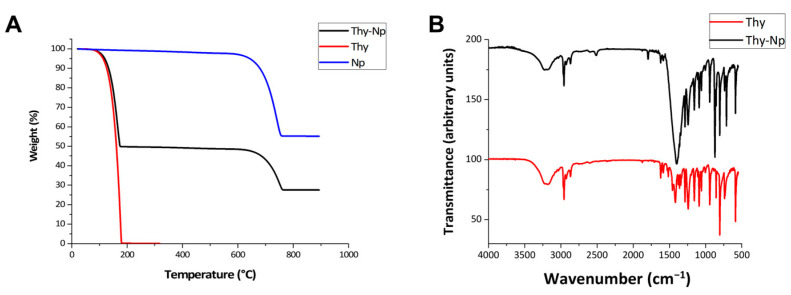
(**A**) TGA thermograms of thymol-loaded CaCO_3_ nanoparticles (black trace) of the as-prepared CaCO_3_ nanoparticles (blue trace) and of pure thymol (red trace); (**B**) FT-IR spectra (transmittance in a.u.) of thymol-loaded CaCO_3_ nanoparticles (black trace) and of pure thymol (red trace).

Thymol capacity loading was confirmed by direct measure, carrying out a TGA analysis on the powdered samples. A typical TGA trace of the thymol loaded material has been reported in Figure 2A (black trace) in comparison with nanoCaCO_3_ (blue trace) and pure thymol (red trace). The pure thymol thermogram shows the expected loss by evaporation, which is complete at 175 °C; on the other hand, two clear thermic events can be observed in the thermogram of the Thy-Np samples; the first, which is correlated to the above mentioned loss of physiosorbed thymol, occurring at the same temperature of the pure sample (red trace), followed by the expected CaCO_3_ calcination event, occurring at ca. 700 °C, was confirmed by comparison with the thermogram of nanoCaCO_3_ (blue trace).

The weight loss in the first thermal event is ca. 50.2%, suggesting an analogous amount of adsorbed thymol in the Thy-Np sample. This experiment is in agreement with the loading capacity of ca. 0.9 mg of thymol/1 mg of nanoCaCO_3_, estimated by the indirect method (see Table 1). The binding of the active principle to the inorganic support can be supposed, as warranted by physisorption. Thymol total desorption, in fact, occurs at temperatures well below its boiling point. Moreover, the FT-IR spectrum of pure thymol (Figure 2B, red trace) has been compared with that of Thy-Np (Figure 2B, black trace). As expected, the latter contains the expected intense and broad carbonate absorption bands centred at ca. 1400 cm^–1^, as well as a sharp absorption at ca. 872 cm^–1^. However, a comparison of the FT-IR spectra of pure thymol and Thy-Np in the remaining section of the IR fingerprint region (see Figure 2B) reveals thymol absorption bands, showing very similar relative intensities at almost superimposable wavenumbers, supporting the fact that the thymol structure is practically unperturbed upon adsorption (physisorption).

The *X. fastidiosa* pathogenic effect is due to invasion and obstruction of xylematic vessels, as just discussed. Therefore, the contrast of this pathogen usually provides two types of phyto-drugs administration: fertigation and endotherapy. We previously verified nanoCaCO_3_ roots adsorption and translocation in olive cuttings and seedlings, which are exposed to nanocarrier aqueous suspensions. Furthermore, we set a protocol for the administration of phytodrugs-loaded nanoCaCO_3_ to olive infected plants through irrigation under greenhouse conditions [28]. In this context, we set up the antibacterial studies of Thy and Thy-Np. Aqueous bacterial suspensions (water for fluorometric test and PYE broth for in vitro inhibition assay) were studied in line with biocide administration and its target site (plants xylema).

### 3.2. Antibacterial Activity of Thy and Thy-Np on X. fastidiosa

**Figure 3:**
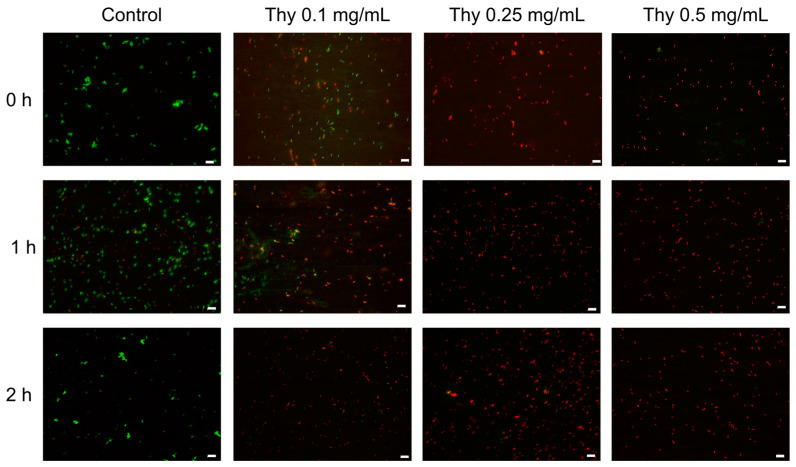
Fluorescence microscopy images of XfDD cells in water suspensions after 0–1–2 h of exposure to thymol solutions at 0.1–0.25–0.5 mg/mL. The control is non-treated cells. Scale bar is 10 µm. Representative images from three experiments.

Thymol is widely recognised as an antibacterial compound. However, there are not yet data about its effect on *X. fastidiosa*. Maddox et al. investigated some phenolic compounds on the *X. fastidiosa* strain *Temecula*; caffeic acid, catechin, p-coumaric acid, resveratrol, rutin and sinapic acid catechol, coumarin, and gallic acid inhibited in vitro *X. fastidiosa* growth [24]. Several works investigated antibacterial action of thymol on foodborne pathogens, human pathogens, and plant pathogens, suggesting its wide use as a biocide [16,22,23]. First, we have investigated the potential use of thymol on water suspensions of XfDD cells by a fluorescence microscopy kit for monitoring bacterial populations’ viability as a function of membrane integrity. Cells with a compromised membrane, which are considered non-vital, were stained in red by the propidium iodide fluorocrome that penetrated inside by the disrupted membranes, while the flurocrome Syto 9 is capable to penetrate the intact membranes of live bacteria, staining them in green. Fluorescence microscopic images of control and treated bacteria are reported in Figure 3. Thymol affected cells’ vitality just after a few minutes of exposition at the lower tested concentration. The effect is more evident, increasing both phenol concentration and exposition time. *X. fastidiosa* cells appeared completely dead within 1 h of treatment with Thy at 0.25 mg/mL. This qualitative test confirmed the expected antibacterial effect of thymol, which is known for its membrane destabilization action [12].

**Figure 4:**
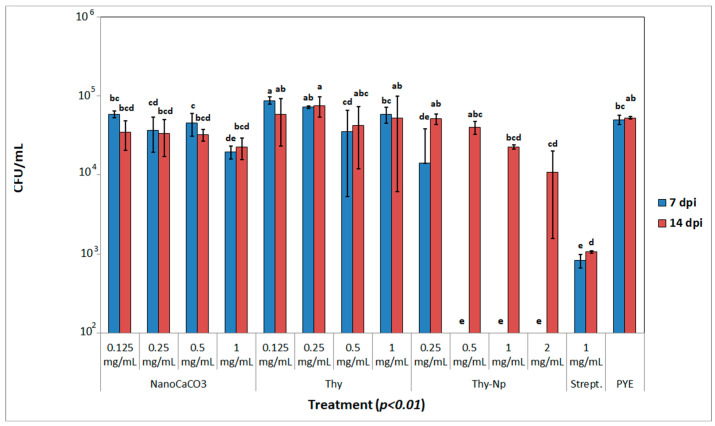
In vitro growth test data (CFU/mL) on XfDD at seven and fourteen days post-inoculation (dpi), following treatment with NanoCaCO_3_, Thy and Thy-Np at different concentrations (range 0.125–2 mg/mL). Streptomycin at 1 mg/mL and PYE broth alone were used as controls. Different letters refer to statistical differences among the treatments after one way analysis of variance (ANOVA), which was performed on the means at each time point. Data are represented as means ± standard deviation. The experiment was repeated twice. No differences among the results of the two independent experiments were noted.

Then in vitro growth test was performed with two cells samples at seven and fourteen days of treatment and comparing free thymol with Thy-Np and empty nanoCaCO_3_. Moreover, streptomycin was used as a positive control (see plot in Figure 4). The tested concentrations of Thy-Np are twice that of Thy and nanoCaCO_3_, as indicated by adsorption capacity data (Section 3.1). This is important to compare samples with the same quantity of biocide.

The most promising results in terms of growth inhibition were showed by Thy-Np when used, starting from 0.25 mg/mL at 7 dpi. Thy and nanoCaCO_3_ had just a slight inhibitory effect on the growth of XfDD at higher concentration (1 mg/mL): indeed, no statistically significant differences were observed among the lower concentrations of Thy and NanoCaCO_3_, indicating that a synergic effect could be achieved by Thy-Np administration. During the whole experiment, a strong dose-dependent effect was observed for each tested substance. At 7 dpi, the amount of detected XfDD CFU/mL in Thy-Np samples was the lowest and comparable to the one showed by the streptomycin control. The same trend was also observed at 14 dpi: the higher concentration of tested Thy-Np (2 mg/mL) exhibited an effect still comparable to streptomycin, while, in all other theses, the bacterial populations were able to grow again. Albeit, the population levels detected at 14 dpi at the higher concentration of the Thy-Np were the lowest, and they have been increasing during time, pointing out that thymol could have a major role in inhibiting the bacteria in the early interactions. These results are compatible with the observations made with the live/dead cell viability assay: Thy alone has been shown to be able to inhibit most of the bacterial cells after a short exposure time (0–2 h), but, after a long period (seven days and more), the inhibition assay has revealed the loss of most of its antimicrobial properties. Therefore, the combination with a nanocarrier could have shielded Thy from degradation and deposition, preserving its biological properties for a longer period, even through a smart delivery mechanism. In this sense, more studies are needed to better understand the antibacterial properties of Thy in relationship with the *X. fastidiosa* biology.

These data suggested a synergic effect of phenol and nanocrystals. This mechanism is supported by previous reports of thymol and other phenols bioactivity and by our results about nanoCaCO_3_ interaction with *X. fastidiosa* (strain *De Donno*) cells [12,24,28].

Probably, Thy-Np caused great membrane depolarization, which not only allowed cell uptake, but also killed bacteria at the lowest concentrations compared to free biocide [39]. In fact, the poor water solubility of thymol is a key limiting factor in relation to its bioavailability and activity at high concentrations. Kumari et al. have found a very significant antibacterial and disease control on *Xanthomonas axonopodis pv*. glycine of soybean, improving thymol bioavailability trough surfactants-based nanoemulsions [21].

Previous ultrastructural analysis demonstrated that nanoCaCO_3_ affected *X. fastidiosa* bacterial wall integrity, and this mechanical effect could drive chemical action of thymol, allowing its uptake in target cells [28]. Therefore, the nanoCaCO_3_ choice was functional to increase thymol bioavailability.

## 4. Conclusions

Recently, thymol-based micro/nano spheres and nanoemulsions have been developed and experimented as antibacterial tools in biomedical, cosmetical, food preservation, and in crop production. These studies demonstrated the great potential of thymol nanoformulations in several applications fields, including agriculture [16,22].

Our results are the first about thymol effect on *X. fastidiosa* conducting tests on the strain “*De Donno*” ST53 of the subspecies *pauca*, the causal agent of olive quick decline syndrome.

First, a fluorometric assay demonstrated the lethal effect of thymol after a few minutes of exposition on water suspensions of XfDD cells, already at the lowest tested concentration. These data confirmed the great potential of phenol as a biocide.

Then, we successfully adsorbed thymol on the nanoCaCO_3_ surface, and we also demonstrated both by direct and indirect quantification, obtaining the Thy-Np nanoformulation.

An in vitro growth test was performed at seven and fourteen days post-inoculation, following exposition to free thymol, which was compared to Thy-Np and nanoCaCO_3_. Free thymol seemed to lose its inhibition effect during bacterial growth. Instead, a strong growth inhibition effect was observed after 7 dpi, already at 0.5 mg/mL of nanoformulation. This bactericidal effect is due to the synergic mechanism of nanoCaCO_3_ mechanical action and thymol chemical effect on *X*. *fastidiosa* cells homeostasis. Thymol is poorly soluble in water, and its free administration could be not very effective. Instead, Thy-Np formulation increased its bioactivity thanks to the delivery action of nanocarriers. Previous data demonstrated that nanoCaCO_3_ affected *X*. *fastidiosa* wall integrity, and this mechanical effect could improve thymol bioavailability, allowing its uptake and action into pathogens cells [28]. Further studies are now ongoing to evaluate the effectiveness of the proposed compound in a controlled environment and field context, also taking into account the possible desirable or less desirable effects on the physiology of the host plant.

## Figures and Tables

**Table 1:** Adsorption efficiency and capacity (mean ± standard deviation) from five replicates for each condition on 100 mg of nanoCaCO_3_ mixing at RT o.n. in water/ethanol solutions (ratio 4/1).

[Loading Solution]	AE	AC
2 mg/mL	-	-
5 mg/mL	20 ± 1.6%	-
25 mg/mL	90.7 ± 0.95%	95.4 ± 5.5%

**Table 2:** Average diameter ± standard deviation of dried Thy-Np by SEM images analysis and average hydrodynamic diameter ± standard deviation and PDI of Thy-Np water suspensions by DLS. Image analysis was made on 100 Np and DLS data, which were obtained by mean of three measurements, each constituting 14 runs.

SEM Measurement (nm)	DLS Measurement (nm)	PdI
82 ± 22	800 ± 0.1	0.8

## Data Availability

All data are available in the manuscript.
